# Technical efficiency of Shiraz school of medicine in research and education domains: a data envelopment analysis

**Published:** 2016-01

**Authors:** SOMAYEH DELAVARI, RITA REZAEE, NAHID HATAM, SAJAD DELAVARI

**Affiliations:** 1 Department of Medical Education, School of Medical and Center for Educational Research in Medical Sciences(CERMS), Iran University of Medical Sciences, Tehran, Iran;; 2 Student Research Committee, Shiraz University of Medical Sciences, Shiraz, Iran;; 3 Quality Improvement in Clinical Education Research Center, Education Development Center, Shiraz University of Medical Sciences, Shiraz, Iran;; 4 Medical Management and Information School, Shiraz University of Medical Sciences, Shiraz, Iran;; 5 Department of Health economics and management, School of Public Health, Tehran University of Medical Sciences, Tehran, Iran

**Keywords:** Education, Medical School, Efficiency, Resource Allocation

## Abstract

**Introduction:**

Efficiency evaluation of universities and faculties is one of the tools that help managers to identify the departments’ strengths and weakness. The main objective of the present research was to measure and compare the technical efficiency of Shiraz school of medicine departments using Data Envelopment Analysis (DEA) technique.

**Methods:**

This cross-sectional and retrospective study was performed on clinical and non-clinical departments in research and education domains over the period of 2006 to 2011. Different inputs and outputs were considered for research and educational domain separately. Efficiency was measured based on the observed optimal performance.

**Results:**

Findings showed that pathology and anatomy departments achieved the score of 100 in technical efficiency in education during 2006 to 2011. During this period, parasitology, psychiatric and pediatrics department’s achieved the score of 100 for technical efficiency in research domain. The lowest mean of relative educational efficiency belonged to orthopedic department; as to relative research efficiency, the lowest mean was shown in orthopedics and genetics departments. The mean technical efficiency of non-medical departments in education and research domain was 91.93 and 76.08, respectively, while the mean technical efficiency of the clinical department in educational and research fields was 91.02 and 82.23, respectively.

**Conclusion:**

Using multiple input and output in DEA technique provided a comprehensive evaluation of efficiency in Shiraz school of medicine departments. The DEA could successfully estimate the technical efficiency of the departments in research and educational fields. Moreover, the deficiency in each department was found; this could help them to plan for improvement.

## Introduction

Mankind is always trying to increase the volume of production and make the best use of facilities and resources to boost the efficiency and productivity (1). The main subject in organizational analysis is their efficiency and their improvement needs to be measurement. Thus, there is no successful organization or university without an efficiency evaluation system (2), since with lack of information about goal achievement, other functions of the organization, such as feedback and detection of weaknesses, are impossible. Thus, improvement and awareness is critical for organizational success (3). Existence of an effective and efficient efficiency evaluation system is of great significance for each organization, such as a university (2).

In universities, managers are always under pressure to improve the performance of educational departments (4). Evaluation systems are based on the notion that anything that is not measurable cannot be monitored and managed (1, 3, 5).

Efficiency evaluation of universities and schools is one of the tools for effective management which helps to identify weaknesses and strengths of each department (6-8). The main objective of efficiency evaluation is to provide real time feedback for making proper modification in order to improve performance. Thus, implementation of evaluation and measurement of the efficiency of the system plays a significant role in improving universities’ output (8, 9).

In this field, each university should use a scientific pattern of efficiency evaluation to facilitate measuring the output of activities. One of the most useful tools in this domain is data envelopment analysis (DEA) technique (5). DEA is one of the most important tools for efficiency measurement which directly uses observable data (7).

Data envelopment analysis is a non-parametric mathematical technique which is based on a linear model. This, based on cross-sectional observation in a specific period of time, could measure the efficiency of strategic business units of an organization via multiple inputs and outputs (1, 3, 9-12). Since in using DEA the change in efficiency is a result of change in the number of input and output as well a change in strategic business unit, the technique measures the relative efficiency (13). Measuring relative efficiency of strategic business units could aid in decision making process which results in selecting the best advice for achieving the organizational goals (7).

DEA as an efficient technique can recognize inefficiency of a unit in comparison with other similar efficient units (14, 15) and accordingly the rate and source of inefficiency will be detected for each department separately (16).

Several studies have been performed for measuring university’s efficiency in both developing and developed countries. Rahimian and Soltanifar (2013) (17), Lopez and Lanzer (2002) (18), Abdulkareem and Oyeniran (2011) (3), Daneshvar and Erol (2009) (7), Shaikh Awadz (2012) (19), Wei and Ahmad (2012) (20) used DEA for efficiency evaluation in research and education field of developing countries. On the other hand, Green and Tomkins (2008) (8), Zheng and Stewart (2002) (21), Sav (2013) (22), Lee (2011) (23), Warning (2004) (24) used DEA in developed countries to evaluate the university’s efficiency. The results of these researches showed that DEA was capable of finding the rate and source of inefficiency of universities.

Inefficient organizations waste valuable substantial resources. DEA can differentiate between efficient and inefficient departments even in small samples. This can direct universities to realize their weaknesses and take appropriate measures for improvement. Furthermore, this can help them to find out the best way of achieving strategic goals. 

With respect to the advantage of DEA in selecting efficient departments as a comparison criterion for inefficient ones, the technique can guide the departments to create strategic action plan for improvement. Finally, it can assist the university administrators in allocation of suitable resource. The above-mentioned facts about DEA justify its use for better managing educational departments of universities.

With respect to advantages of DEA, the present research aimed to use DEA to evaluate educational and research efficiency of Shiraz school of medicine, which is one of the pioneer and best medical schools in Iran.

School of medicine is the most important school in Shiraz University of Medical Sciences. Since it has the most number of professors and students, as well as diversity of disciplines, it was selected as the study setting. Measuring efficiency in this school could provide valuable information for managers and decision makers. DEA could identify efficient and inefficient departments of the school of medicine and also help the head of the departments to detect their weaknesses and use the efficient departments as their model. This also can make a sense of competition among departments toward improvement. 

## Methods

This is a cross-sectional and retrospective study which was performed for measuring the efficiency of Shiraz school of medicine different departments using DEA technique over the period of 2006-2011. In this study, 19 clinical and 10 basic sciences departments were selected as decision making units. Since, the data were gathered from documents and reports; there was no sampling and no statistical error. Data were gathered from vice chancelleries of education and research of Shiraz University of Medical Sciences. 

With regards to the differences in the nature of departments at Shiraz school of medicine, they were divided into two categories (clinical and basic sciences). Thus, ten departments were assigned in basic sciences and nineteen departments in clinical sciences. Clinical departments included ENT, radiotherapy, radiology and nuclear medicine, orthopedics, surgery, cardiology, urology, neurology, neurosurgery, psychiatrics, pediatrics, gynecology and obstetrics, rehabilitation, dermatology, anesthesia, ophthalmology, internal medicine, pathology, and community medicine. And the basic sciences departments studied were pharmacology, biochemistry, parasitology, genetics, medical physics, bacteriology, physiology, anatomy, biostatistics, and immunology.

After categorization, inputs and outputs for both categories were selected separately. For this, inputs and outputs were extracted from the literature. Then, two focus group discussions were held with department heads to finalize the criteria of inputs and outputs.

The number of master and Ph.D degree students, number of faculty members with Ph.D degree (professors, associate professors, and assistant professors), and number of instructors were assumed as inputs for basic sciences departments. The number of graduates from master degree (T1), number of graduates from Ph.D degree (T2), number of written or translated books by faculty members (T3), courses which were taught by faculty members of a department to students in other related majors (T4), and number of promoted faculty members (T5) were considered as outputs for basic sciences departments in education domain.

In clinical departments, the number of residents, fellowships, and faculty members (professors, associate professors, and assistant professors) was regarded as inputs in education domain. Outputs of clinical departments included the number of graduates from specialist and subspecialist degree (T1), number of top rank students in the board exam (T2), number of graduates who received the board degree (T3), number of written or translated books by faculty members (T4), and the mean scores of practical board exam (T5) and theoretical board exam (T6).

Four inputs were assigned for research domain similar in both basic science and clinical sciences. These were the total number of faculty members (with master and Ph.D degree), number of students, number of research centers founded by the faculty members of a department. Outputs were the numbers of published papers indexed in ISI or PUBMED (R1), number of published papers in other Persian and English journals (R2), papers presented in national and international conferences (R3), completed dissertations (R4), approved research projects (R5), and finished research projects (R6).

After data collection, in the third phase, data were entered into MS excel and DEA master software and analyzed using Data Envelopment Analysis technique. Technical efficiency for both clinical and basic sciences departments was estimated based on output oriented and variable return to scale (VRS) assumption. Then, lack of outputs for each department was determined separately. Deficiencies show changes that departments should make in order to reach optimum efficiency (E=100) in comparison with efficient departments. 

It worth mentioning that the departments’ efficiency was calculated based on the best performance in comparison with other departments. Thus, the measured efficiency was relative when a department was compared with another department with similar inputs and outputs. Those groups that had efficiency rate of 100 were regarded as efficient departments and those obtaining a rate lower than this were inefficient. Thus, the more the inefficiency rate, the more wastefulness of resources in departments. Since, efficiency measurement was output-oriented; lack of output shows the amount of changes in outputs that department heads should make without any change in inputs in order to reach the maximum efficiency. 

## Results

As a result of data analysis, technical efficiency of clinical and basic sciences departments in research and education domains was measured and their deficiencies were determined. Finally, the mean score of these departments was compared in each year separately. At first, the results of efficiency measurement in education and then in research domain are presented.

Table 1 shows the mean score of education efficiency and lack of outputs in basic sciences departments. The scores are sorted from highest to lowest. According to Table 1 in education domain, anatomy was the only department that scored 100 in technical efficiency in all six years and the lowest score of efficiency belonged to parasitology department. The mean efficiency score in basic sciences departments was 91.93 among inefficient departments, ranging from 75.93 to 99.37.

**Table 1:** Technical efficiency and mean lack of each output for basic sciences departments in education over the period of 2006 to 2011

**Departments**	**Mean technical efficiency±SD**	**Mean lack for each output**				
		T1	T2	T3	T4	T5
**Anatomy**	100±0	0	0	0	0	0
**Biostatistics**	99.37±1.53	0	0	0	0.24	0
**Medical Physics**	97.85±3.74	0	0	0	1.9	0
**Bacteriology**	96.64±4.73	0.04	0	0	1.98	0
**Genetics**	95.55±6.88	0	0	0	0.66	0
**Pharmacology**	94.13±14.36	0	0	0	3.16	0
**Biochemistry**	90.74±10.44	0.12	0.06	0	5.22	0.03
**Physiology**	89.8±12.73	0.25	0.03	0	9.26	0.12
**Immunology**	79.27±32.12	0.52	0	0	16.47	0
**Parasitology**	75.93±22.33	0.60	0.14	0	14.88	0.32
**Average**	91.93					

T1: Number of graduates from master degree, T2: Total number of graduates from PhD degree, T3: Total number of written or translated books by faculty members, T4: Total courses that were taught by faculty members of department to all the students in other related fields, T5: Total number of promoted faculty members

As it can be seen in Table 1, the highest deficiency rate was related to the courses taught by faculty members of a department to the students in other related fields of study (T4), especially in immunology department. This output requires improvement in order to reach its optimum level of efficiency. Hence, anatomy was the only department that did not need any changes. Therefore, anatomy department is the best benchmark for other departments.  In order for parasitology to achieve the highest level of efficiency, the number of graduates in master (T1) and Ph.D degrees and total number of promoted faculty members have to increased.

Table 2 presents the details of the mean score of efficiency and lack of output for clinical department from 2006 to 2011. The efficiency scores are sorted from highest to lowest. As seen in Table 2, among clinical departments, pathology was the only department that achieved the score of 100 in all six years in education domain; however, others did not reach 100. The lowest score among clinical departments belonged to orthopedics. The mean score of clinical departments in education was 91.02 during the mentioned years. The mean efficiency score of inefficient departments varied from 80 to 99.34.

**Table 2:** Technical efficiency and mean lack of each output for clinical departments in education over the period of 2006 to 2011

**Department**	**Mean technical efficiency±SD**	**Mean lack for each output**					
		T1	T2	T3	T4	T5	T6
**Pathology**	100±0	0	0	0	0	0	0
**Pediatrics**	99.45±0.85	0.09	0.05	0	0	0.60	0.65
**Radiotherapy**	97.94±5.03	0.02	0	0	0	2.34	0
**Gynecology**	97.53±2.83	0.18	0.10	0.02	0	2.19	2.87
**dermatology**	96.27±2.64	0.16	0.11	0	0	4.58	4.76
**Community Medicine**	95.31±5.34	0.10	0.08	0.01	0	5.66	5.71
**Rehabilitation**	95.38±7.98	0.18	0.09	0	0	3.81	5.70
**Internal Medicine**	93.92±4.64	1.01	0.50	0.01	0.03	6.31	7.54
**Cardiology**	93.54±6.66	0.53	0.22	0.02	0	8.32	8.39
**General Surgery**	90.41±6.14	1.11	0.68	0	0.63	11.84	11.88
**Neurology**	90.13±4.35	0.46	0.32	0	0.01	12.43	11.79
**Ophthalmology**	88.69±5.86	0.69	1.68	0.06	0.04	13.71	14.66
**Psychiatry**	89.12±9.00	0.67	0.17	0	0.12	12.04	12.44
**Urology**	85.07±7.12	0.47	0.28	0	0	15.98	16.18
**Neurosurgery**	86.84±11.09	0.40	0.22	0.04	0	17.33	8.32
**Anesthesiology**	85.50±6.71	1.53	0.81	0.02	0	17.94	21.65
**E.N.T**	81.41±5.43	0.95	0.25	0	0.02	23.52	23.84
**Radiology**	80.45±8.84	1.12	0.49	0.01	0	23.38	23.15
**Orthopedics**	80±5.93	1.2	0.54	0.06	0.08	26.20	26.67
**Average**	91.02						

T1: The number of graduates from specialist and subspecialist degree, T2: Number of top ranked students in board exam, T3: Number of graduates who received the board degree,T4: Number of written or translated books by faculty members, T5: Mean score of practical board exam, T6: The mean score of theoretical board exam.

As shown in Table 2, the mean score of practical board exam (T5) and the theoretical board exam (T6) were the main sources of inefficiency in all departments. Lack of graduates from specialty and subspecialty degree (T1) and number of top ranked students in board exam (T2) belonged to anesthesia and ophthalmology department, respectively. Furthermore, lack of written or translated books by the faculty members (T4) was highest in general surgery department. In addition, orthopedics department had the lowest mean scores of the practical board exam (T5) and theoretical board exam (T6), and also the number of top ranked students in the board exam (T2). As it was mentioned, pathology was the only department that needed no change in its outputs.

Table 3 shows the mean of technical efficiency and lack of each output in research domain of basic sciences departments. As shown in this Table, parasitology was the only department that could reach the highest rate of efficiency in all six years. The lowest means of efficiency among basic sciences in research domain belonged to the genetics department. The means of efficiency for all basic sciences departments was equal to 76.08. The mean scores of inefficient departments varied from 50.64 to 96.436.

**Table 3:** Technical efficiency and mean lack of each output for basic sciences departments in research over the period of 2006 to 2011

**Departments**	**Meantechnical efficiency±SD**	**Mean lack for each output**					
		R6	R5	R4	R3	R2	R1
**Parasitology**	100±0	0	0	0	0	0	0
**Immunology**	96.43±5.67	0.97	0.79	0.60	0.79	1.17	0.34
**Biostatistics**	92.39±17.29	0.93	0.29	0.15	0.29	1.12	0.26
**Medical Physics**	86.14±22.11	0.74	0.24	0.16	0.24	0.98	0.41
**Bacteriology**	77.36±13.91	2.34	2.72	0.66	2.72	6.18	1.43
**Pharmacology**	73.53±16.20	7.45	3.14	0.92	3.14	4.20	1.26
**Anatomy**	70±12.94	4.25	2.41	1.78	2.41	7.91	2.99
**Biochemistry**	58.1±19.51	3.19	6.33	2.89	6.33	10	4.50
**Physiology**	56.22±23.55	3.47	6.31	4.42	6.31	3.85	5.45
**Genetics**	50.64±25.88	1.43	2.62	0.53	2.62	4.50	0.57
**Average**	76.08						

R1: Total number of published papers indexed in ISI and PUBMED, R2: Total number of published paper in other Persian and English journals, R3: Total number of paper presented in national and international congress, R4: Total number of finished thesis, R5: Total number of approved research projects, R6: Total number of finished research projects

According to Table 3, lack of papers published in journals indexed in ISI or PUBMED (R1) was the highest in pharmacology department. Furthermore, lack of published paper in Persian and English journals (R2), papers presented in national and international conferences (R3), and completed research projects (R6) was related to pharmacology department. On the other hand, lack of completed dissertation (R4) and approved research projects (R5) was highest in biochemistry department. As it was mentioned, according to the analyzed data, parasitology was the most efficient department and did not need any change.

Based on information presented in tables 1 and 3, it can be concluded that anatomy which was the leading educational department was not successful in research. On the other hand, parasitology department which was not successful in education achieved the highest level of efficiency in research domain. Furthermore, immunology department was more successful in research than education and other departments were the opposite.

Table 4 shows the means of efficiency and lack of output for each clinical department. As shown, pediatrics and psychiatrics departments reached the highest level of efficiency in all six years. The lowest means of efficiency in research domain was related to orthopedics department. The mean score of inefficient departments varied from 47.86 to 98.771.

**Table 4:** Technical efficiency and mean lack of each output for clinical departments in research over the period of 2006 to 2011

**Departments **	**Mean technical efficiency±SD**	**Mean lack for each output**					
		R6	R5	R4	R3	R2	R1
**Psychiatry**	100±0	0	0	0	0	0	0
**Pediatrics**	100±0	0	0	0	0	0	0
**Pathology**	98.77±3.01	0.82	0.18	0.13	0.14	0.15	0.21
**E.N.T**	98.36±2.82	0.32	0.09	0.17	0.20	0.03	0.07
**Community Medicine**	94.96±9.57	0.37	0.34	0.63	0.60	0.17	0.50
**Internal Medicine**	94.57±6.68	3.62	1.15	1.61	2.17	1.72	1.86
**General Surgery**	90.08±8.42	6.44	1.41	1.92	2.74	4.45	1.55
**Radiotherapy**	89.50±16.73	0.96	0.80	0.32	0.31	1.24	0.11
**Dermatology**	88.23±20.13	2.76	0.50	1.05	1.84	1.41	0.13
**Radiology**	84.50±18.20	4.61	0.88	2.7	3.00	0.64	0.75
**Rehabilitation**	83.75±17.89	1.24	1.46	1.74	1.99	2.06	1.70
**Cardiology**	80.67±21.52	8.72	3.28	3.99	2.97	1.37	2.56
**Anesthesiology**	80.47±11.36	2.92	2.41	3.63	5.10	3.24	1.43
**Ophthalmology**	78.73±20.52	10.06	1.77	4.02	5.79	3.17	2.44
**Gynecology**	78.40±14.05	5.47	2.30	4.62	4.93	3.31	2.72
**Neurology**	67.91±15.47	8.63	3.53	3.33	4.67	3.29	1.87
**Urology**	57.71±24.62	8.33	4.10	5.28	6.26	6.71	3.15
**Neurosurgery**	49.59±10.37	8.75	2.28	4.13	6.16	5.16	2.25
**Orthopedics**	47.86±22.68	12.18	4.86	7.89	6.80	5.93	4.22
**Average**	82.33						

R1: Total number of published papers indexed in ISI and PUBMED, R2: Total number of published paper in other Persian and English journals, R3: Total number of paper presented in national and international congress, R4: Total number of finished thesis, R5: Total number of approved research projects, R6: Total number of finished research projects

According to Table 4, lack of papers published in journals indexed in ISI or PUBMED (R1), published papers in Persian or other English journals (R2), presentations in national or international conferences (R3), number of completed desertions (R4), and number of completed research projects (R6) were the highest in orthopedics department. On the other hand, lack of approved research projects (R5) was the highest in urology department. Pediatrics and psychiatrics did not need any change in output.

If the results of tables 2 and 4 are compared with each other, psychiatrics, pediatrics, ENT, internal medicine, and radiology were more successful in research than education and vice versa in other clinical departments. Also, the difference between efficiency scores in research and education for internal medicine, urology, neurosurgery, and orthopedics departments was noteworthy.

## Discussion

Universities play an important role in development of a country. Thus, faculties and departments should pursue a set of goals which makes them more efficient. Thus, they should be monitored using a set of criteria and, accordingly, some measures should be taken to modify inefficient department into efficient ones. For these reasons, technical efficiency of the departments in Shiraz School of Medicine was measured and their needs for change were determined in this study. 

DEA technique, a useful tool for a precise comparison of efficiency between different departments which was impossible before using this technique, accomplished a comprehensive evaluation of Shiraz School of Medicine departments. For the first time, efficiency was measured in 10 basic medical sciences and 19 clinical departments over a six year period. 

Results revealed that different types of determining strategies by data envelopment analysis could assist in attaining educational and research goals by providing suitable information (21). Data envelopment analysis is a flexible technique which can meet the policymakers’ needs for measuring inputs and outputs (24). Using the results of the present research provides precise information about the departments’ technical efficiency. Through this, controlling and allocating resources to departments will be more accurate and the school will move toward improvement (7). Measuring technical efficiency allows managers to ask for more resources and facilities based on their improvement (21). The results could help the faculty real improvement in resource management.

Generally, it seems that technical efficiency of clinical and basic sciences departments in educational domain are higher than research. Results also revealed that departments’ performance in education is homogenous. Nevertheless, it cannot be concluded that these departments are efficient or inefficient when they are compared with those in other school. Efficiency score calculated by DEA is relative and it can change according to change in inputs and outputs as well as change in the number of departments. Thus, when efficiency score is equal to 100, it cannot be deduced there is no need for improvement. 

Results revealed that if a department has a wider gap with the score of 100, more improvement is required in the output. Data envelopment analysis could determine the optimum rate of each output in order to achieve the score of 100 for each department separately. Thus, it can help the departments in the school of medicine to find their weaknesses and plan for further improvement (18-24).

The results are applicable for departments to make their plan for change clear and also give them a vivid perception about their goals and objectives. According to the findings, increase in potential outputs (total courses that were taught by faculty members of departments to all the students in other fields, mean score of practical and theoretical board exam for clinical departments, total number of published papers indexed in ISI or PUBMED, number of completed dissertations, and number of approved research projects) could have a significant impact on departments’ efficiency. 

The present study revealed that efficiency measurement by using data envelopment analysis is a suitable method for evaluation of efficiency in universities and schools because it considers several inputs and outputs (21). Data envelopment analysis can help departments to find the best path for achieving strategic goals (10). This could create a sense of competition among departments that result in improvement and promotion of the university. Sense of competition in school could increase the quantity and quality of research and educational activities and make a better image of the university in the international level.

## Conclusion

Since universities are compared in national and international level in research and education domains, it is suggested that efficiency of schools and universities should also be compared with each other.

 According to our results, in order to measures the efficiency of a university, at first we should determine the goal of university, and the stakeholders’ responsibility should be determined. The university should be provided with valid and comparable data in different levels that can adjust the results based on each university characteristics. Data gathering and analysis should be according to scientific methods and imperative. Criteria should be noted in research and education domain. 
